# Pokémon GO and Physical Activity in Asia: Multilevel Study

**DOI:** 10.2196/jmir.9670

**Published:** 2018-06-15

**Authors:** Ben D Ma, Sai Leung Ng, Tim Schwanen, John Zacharias, Mudi Zhou, Ichiro Kawachi, Guibo Sun

**Affiliations:** ^1^ Department of Geography and Resource Management The Chinese University of Hong Kong Hong Kong China (Hong Kong); ^2^ Institute of Space and Earth Information Science The Chinese University of Hong Kong Hong Kong China (Hong Kong); ^3^ Transport Studies Unit School of Geography and Environment University of Oxford Oxford United Kingdom; ^4^ College of Architecture and Landscape Peking University Beijing China; ^5^ Department of Mathematics Imperial College London London United Kingdom; ^6^ Department of Social and Behavioral Sciences TH Chan School of Public Health Harvard University Boston, MA United States; ^7^ Department of Urban Planning and Design Faculty of Architecture The University of Hong Kong Hong Kong China (Hong Kong)

**Keywords:** physical activity, Pokémon Go, public health intervention, exergame, weather

## Abstract

**Background:**

Physical activity has long been considered as an important component of a healthy lifestyle. Although many efforts have been made to promote physical activity, there is no effective global intervention for physical activity promotion. Some researchers have suggested that Pokémon GO, a location-based augmented reality game, was associated with a short-term increase in players’ physical activity on a global scale, but the details are far from clear.

**Objective:**

The objective of our study was to study the relationship between Pokémon GO use and players’ physical activity and how the relationship varies across players with different physical activity levels.

**Methods:**

We conducted a field study in Hong Kong to investigate if Pokémon GO use was associated with physical activity. Pokémon GO players were asked to report their demographics through a survey; data on their Pokémon GO behaviors and daily walking and running distances were collected from their mobile phones. Participants (n=210) were Hong Kong residents, aged 13 to 65 years, who played Pokémon GO using iPhone 5 or 6 series in 5 selected types of built environment. We measured the participants’ average daily walking and running distances over a period of 35 days, from 14 days before to 21 days after game installation. Multilevel modeling was used to identify and examine the predictors (including Pokémon GO behaviors, weather, demographics, and built environment) of the relationship between Pokémon GO use and daily walking and running distances.

**Results:**

The average daily walking and running distances increased by 18.1% (0.96 km, approximately 1200 steps) in the 21 days after the participants installed Pokémon GO compared with the average distances over the 14 days before installation (*P*<.001). However, this association attenuated over time and was estimated to disappear 24 days after game installation. Multilevel models indicated that Pokémon GO had a stronger and more lasting association among the less physically active players compared with the physically active ones (*P*<.001). Playing Pokémon GO in green space had a significant positive relationship with daily walking and running distances (*P*=.03). Moreover, our results showed that whether Pokémon GO was played, the number of days played, weather (total rainfall, bright sunshine, mean air temperature, and mean wind speed), and demographics (age, gender, income, education, and body mass index) were associated with daily walking and running distances.

**Conclusions:**

Pokémon GO was associated with a short-term increase in the players’ daily walking and running distances; this association was especially strong among less physically active participants. Pokémon GO can build new links between humans and green space and encourage people to engage in physical activity. Our results show that location-based augmented reality games, such as Pokémon GO, have the potential to be a global public health intervention tool.

## Introduction

As a Pokémon GO player, the first author of this paper walked 1526 km over 5 continents (Asia, Africa, Europe, North America, and South America) during the course of the game. This substantially increased his walking activity. His case may be that of an outlier, but increased walking is very common among Pokémon GO players. Pokémon GO is a location-based augmented reality (AR) game, which was launched in July 2016. Impressively, it receives 65 million monthly active players and has recorded more than 650 million app downloads within 6 months [1]. Pokémon are virtual creatures that inhabit the fictional Pokémon World. Pokémon GO allows players to locate, capture, and battle Pokémon on their mobile devices, as if the Pokémon were in the same real-world locations as the players. As players move around their real world, their avatars in the game move within the in-game map based on real world geographical locations. The in-game map contains many features such as “Gyms” and “Pokéstops,” where players can get Poké balls and other items to catch and battle with Pokémon. To play this game, players need to move around in the real world and not be sedentary. By May 2017, 9% of the global population installed Pokémon GO and traveled more than 15.8 billion km while playing Pokémon GO [2].

In this study, we sought to measure the relationship between Pokémon GO use and the players’ physical activity and how this relationship varied across players. Physical activity has long been considered an essential component of a healthy lifestyle [3-5]. Although many efforts to promote physical activity [5-7] have been undertaken, there is no effective global intervention for physical activity promotion [8]. Physical inactivity is the second-leading cause of preventable death worldwide, next to smoking [5].

The literature indicates that physical activity is affected by various variables, such as demographics [9-11], body mass index (BMI) [12,13], weather [14-16], and built environment [17-19]. Recently, increasing research suggested that new technologies, especially “gamified” physical activity interventions, are used to promote physical activity [20]; however, it was not until 2016 that Pokémon GO provided the first glimpse of how to intervene for the promotion of physical activity on a global scale.

Past studies found that Pokémon GO was associated with short-term increases in players’ physical activity levels. The existing literature can be mainly divided into 2 groups. Objectively-collected physical activity data indicated that there was an increase in the daily number of walking steps among the Pokémon GO players after game installation [21,22]. Studies using self-reported data indicated that players spent more time outside and performed more physical exercise because of Pokémon GO [23-26]. Other research included studies on Pokémon GO players’ motivations [27,28], experiences while playing Pokémon GO [29], and potential adverse effects associated with playing Pokémon GO [30,31].

However, there remain 2 major research gaps. First, physical activity on each day is not only determined by players’ demographics and Pokémon GO behaviors (such as whether Pokémon GO was played, and the number of days played) but also by weather and built environment. Inclement or extreme weather can be physical activity barriers [15]. Further, built environment is the essential element for location-based AR games because the games are built based on the actual physical world environment. However, existing research failed to take them into account when investigating the relationship between Pokémon GO use and physical activity.

Second, although some researchers suggested that the association between Pokémon GO and physical activity varies among different populations [24,25,32,33], to date, little research has been performed on these potential differences among players. Howe et al focused on young adults [21]; Althoff et al studied wearable Microsoft product users [22]; Kogan et al focused their study on dog owners [34]; and Wong et al researched university students [26]. Asia is home to 60% of the world’s population [35]; although Pokémon GO has been suspended in Mainland China, there are still considerable numbers of Pokémon GO players in other parts of Asia. However, most studies were conducted in Western countries, and there are no objective studies of Pokémon GO and physical activity in Asia.

Therefore, we used a multilevel modeling approach to answer 2 main research questions. They are as follows:

- What is the relationship between Pokémon GO use and players’ physical activity levels?
- How does the association between Pokémon GO and physical activity vary across players with different physical activity levels?

## Methods

### Research Design

We conducted a field survey in August 2016 during the first month after Pokémon GO was launched in Hong Kong. Researchers approached Pokémon GO players at 5 study sites that represented 5 typical types of built environment. Participants’ data, including sex, age, income, education level, BMI (kg/m^2^), and the start date of Pokémon GO, were collected using a questionnaire. Participants’ physical activity data, specifically daily walking and running distances, were captured from their iPhone “health” app pages by taking pictures of their screens. Eventually, we compiled a data set covering the period from 14 days before and 21 days after the installation of Pokémon GO. Weather data were collected from Hong Kong Observatory, including total rainfall (mm), bright sunshine (hours), mean air temperature (°C), and mean wind speed (km/h).

**Table 1:** Descriptions of study sites.

Study site	Type of built environment	Description
Central	Office area	Land used for administration, or clerical, technical, professional, or other like business activity
Wan Chai	Mixed use area	Land used for mixed uses
Victoria Park	Green space	Integrated park consisting of playgrounds, sitting-out areas or public/mini sports grounds
Wong Tai Sin	Residential area	Land used to for residential accommodations
Causeway Bay	Retail premises	Land used to: (a) sell goods by retail or by retail and wholesale; (b) sell services; or (c) hire goods

The survey was conducted by our researchers on a face to face basis with each participant in the study areas. All participants provided written informed consent to participate prior to survey conduction. Participants received a Pokémon toy as a token of appreciation for completing the study. All data collected were anonymized, and the participants’ names and home addresses were not collected.

### Study Population

We approached Pokémon GO players while they were playing Pokémon GO within the study sites. To ensure the reliability and consistency of data, only iPhone 5s or 6 series users with qualified daily walking and running distances in their iPhone “health” app were included in this study. We excluded individuals who did not complete the questionnaire, were not iPhone 5s or 6 users, and those who were iPhone users but were unable to provide their daily walking and running distances data. We also excluded respondents with insufficient Pokémon GO levels to unlock the primary functions of the game. The primary function of the game was to reach level 5 and gain access to the Gyms.

### Study Sites

We used 5 study sites, which represented 5 typical types of built environment in Hong Kong, namely, a green space, an office area, a residential area, a mixed-use area, and a retail location (Table 1). For each built environment, we selected a 200-m radial zone based on the surrounding land use [36] and set ≥60% of the surrounding land use belonging to that type of built environment as the criterion for the site selection through a GIS platform [37].

### Daily Walking and Running Distances

Daily walking and running distance data represented the daily physical activities of Pokémon GO players. To compare players’ physical activities before and after installation, we used the average walking and running distances each day over a period of 35 days (14 days before to 21 days after game installation). We estimated 95% CI 5.8-6.0 through a bootstrap with 500 resamples of daily walking and running distances. If there were no data on a given day, which meant the player probably did not touch his or her phone at all, or there were problems with the phone, we ignored the data recorded on that day. We excluded 15 observations from the analysis because the data values were empty.

### Multilevel Modeling

Multilevel modeling was employed to investigate the difference of association between Pokémon GO and daily walking and running distances across players. We have multiple observations of daily walking and running distances of the same individual (from 14 days before to 21 days after the game installation); based on this cluster data structure, multilevel modeling was applied to investigate the relationship between Pokémon GO use and physical activity. At observation level, each observation has its own observation-level attributes, including whether Pokémon GO was played, the number of days played, total rainfall (mm), bright sunshine (hours), mean air temperature (degree Celsius), and mean wind speed (km/h). At player level, age, income, gender, education, BMI (kg/m^2^), and built environment variables were added. Further, interdependencies among different player levels were taken into account through multilevel modeling. We transferred parts of variables into dummy variables based on questions that we intended to investigate. MLwiN V.3.0 was used to conduct the multilevel modeling analysis.

### Funding, Ethical Approval, and Data Sharing

This research received no specific grant from any funding agency in the public, commercial, or not-for-profit sectors. This study was approved by Survey and Behavioral Research Ethics Committee at The Chinese University of Hong Kong on August 4, 2016. The statistical code and dataset are available from the corresponding author.

## Results

### Study Populations and Sites

Overall, 210 Pokémon GO players were included in this study; Table 2 shows the profile of the participants. We identified 2964 individuals as Pokémon GO players while they were playing Pokémon GO within the study sites. Of these, 1248 answered our questionnaire and 1028 players were excluded because they were not iPhone 5s or 6 users or were iPhone users but unable to provide their daily walking and running distance data. Additionally, we excluded those who did not complete the questionnaire (n=7) or who were unable to unlock the primary functions of the game (n=3).

**Table 2:** Characteristics of Pokémon GO players (N=210).

Characteristic		Value
Age (years), mean (SD)		26.1 (8.7)
**Age group (years), n (%)**		
	13-17	25 (11.9)
	18-23	67 (31.9)
	24-29	43 (20.5)
	30-35	35 (16.7)
	36-50	21 (10.0)
	>51	2 (1.0)
Female, n (%)		71 (33.8)
**Education, n (%)**		
	High school or lower	70 (33.3)
	College or higher	118 (56.1)
**Monthly income (HKD), n (%)**		
	<5000	65 (31.0)
	5000-10000	12 (5.7)
	10001-15000	41 (19.5)
	15001-20000	30 (14.3)
	20001-30000	18 (8.6)
	>30000	18 (8.6)
**Site, n (%)**		
	Office (Central)	33 (15.7)
	Mixed use (Wan Chai)	48 (22.9)
	Green space (Victoria Park)	46 (21.9)
	Residential (Wong Tai Sin)	55 (26.2)
	Retail premises (Causeway Bay)	28 (13.3)
**Body mass index (BMI; kg/m^2^), n (%)**		
	Underweight (BMI<18.5)	57 (27.1)
	Normal (18.5≤BMI<25)	121 (57.6)
	Overweight/obese (25≤BMI)	16 (7.6)
Daily walking and running distances in 2 weeks before installation of Pokémon GO (km), mean (SD)		5.4 (3.5)

### Daily Walking and Running Distances Before and After the Game Installation

**Figure 1:**
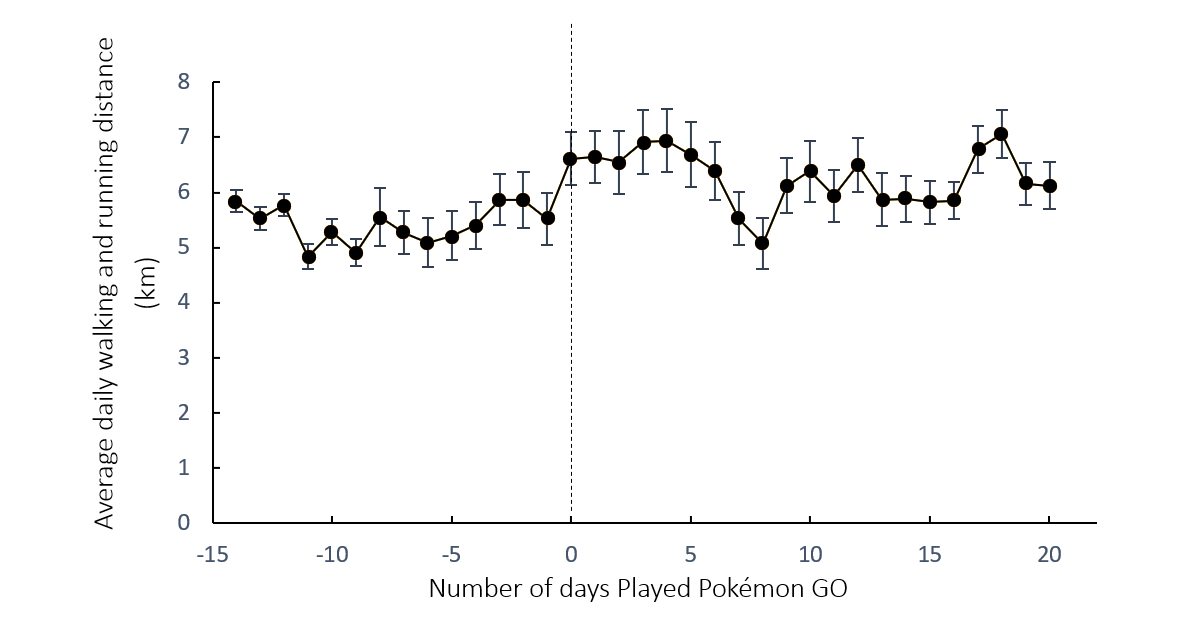
Average daily walking and running distance before and after the installation of Pokémon GO. Error bars correspond to bootstrapped 95% confidence intervals.

Figure 1 shows the change in average daily walking and running distances of the Pokémon GO players in the period from 14 days before to 21 days after game installation. The average distance increased by 18.1% (0.96 km), from 5.30 km (SD 2.12) (before installation) to 6.26 km (SD 2.45) (after installation). A comparison of means shows that Pokémon GO was associated with increases in daily walking and running distances (*F*=33.825, *P*<.001). We observed a decrease in daily walking and running distances during the period from the 5th to 8th day after installation. During that period, Hong Kong’s weather deteriorated because of the influence of the typhoon Nida.

### Pokémon GO and Physical Activity across Players

The results of multilevel models are shown in Table 3. A null model, a model without any input variances, which simply describes the variance at each level, was conducted first. We found that 22.5% of the variance was explained at the observation level, and this provided justification for proceeding to the subsequent analysis.

Model 1 contains observation-level variables. In model 2, the player-level variables were added and analyzed together with the observation-level variables (Table 3). In model 3, assuming that all other variables in the model were held constant, the relation between Pokémon GO behaviors and dependent variables were investigated. In model 4, on the basis of model 3, the interactions of Pokémon GO use (or no Pokémon GO use) and built environment were included (Table 4).

The results of multilevel models showed that the observation-level variables (including whether Pokémon GO was played, the number of days played, total rainfall, bright sunshine duration, mean air temperature, and mean wind speed) were significantly related to daily walking and running distances. Whether Pokémon GO was played (*P*=.005) and bright sunshine duration (*P*=.01) were significantly associated with the increased daily walking and running distances. Conversely, the number of days played (*P*<.001), total rainfall (*P*=.003), mean air temperature (*P*=.01), and mean wind speed (*P*=.04) were negatively correlated with daily walking and running distances. At the player level, our results indicated that players who were male, had high school or lower education, and exhibited normal BMI (kg/m^2^) were likely to have higher daily walking and running distances (Table 4). Furthermore, playing Pokémon GO in green space was associated with higher daily walking and running distances (*P*=.03).

Playing Pokémon GO was significantly associated with increased daily walking and running distances (*P*=.005), whereas the number of days played was significantly associated with decreased daily walking and running distances (*P*<.001). The results showed that the association between Pokémon GO and daily walking and running distances attenuated over time and may have dissappeared after players played Pokémon GO for 24 days (Table 4).

Most importantly, our results indicate that the effects of playing Pokémon GO are somewhat stronger for players with lower levels of daily physical activity and slightly stronger for those who have played the game for fewer days. These findings are obtained when the estimated effects of playing Pokémon GO and number of days played are allowed to vary across individuals in the sample. The negative estimate of the covariance of playing Pokémon GO and intercept (–0.008 in Model 4) captures the stronger health benefits of playing among respondents with shorter walking and running distances. The estimated covariance between days played and whether Pokémon GO was played of −0.00057 shows that those playing the game for a shorter period of time have, all else equal, experienced slightly less health benefits.

**Table 3:** Multilevel models of Pokémon GO and daily walking and running distances (km) models 1 and 2.

Variable			Model 1		Model 2	
			β	*P* value	β	*P* value
**Observation level**						
	Pokémon GO or not^a^ (reference: not)		0.085	<.001	0.084	<.001
	Days played^b^		−0.003	<.001	−0.004	<.001
	**Weather**					
		Total rainfall	−0.001	.013	−0.001	.012
		Bright sunshine duration	0.003	.013	0.003	.013
		Mean air temperature	−0.012	.020	−0.013	.016
		Mean wind speed	−0.001	.010	−0.001	.009
**Player level**						
	**Age (reference >35)**		—	—		
		13-17	—	—	−0.087	.019
		18-23	—	—	−0.016	.320
		24-29	—	—	−0.016	.316
		30-35	—	—	−0.065	.028
	**Income (HKD; reference >30000)**					
		<5000	—	—	−0.011	.385
		5000-10000	—	—	−0.111	.012
		10001-15000	—	—	−0.028	.223
		15001-20000	—	—	0.050	.096
		20001-30000	—	—	0.030	.223
	**Gender (refererence: female)**					
		Male			0.039	.026
	**Education (reference: high school or lower)**					
		College or higher	—	—	−0.077	.036
	**BMI^c^ (reference: normal; kg/m^2^)**					
		Underweight (BMI<18.5)	—	—	−0.069	.004
		Overweight and obese (25≤BMI)	—	—	−0.050	.045
		Built environment (reference: office)	—	—		
		Mixed use area	—	—	0.026	.207
		Green space	—	—	0.041	.094
		Residential	—	—	0.001	.491
		Retail premises	—	—	−0.003	.469
**Intercept**						
	Mean		0.714	<.001	0.735	<.001
	Variance (observation level)		0.058	<.001	0.058	<.001
	Variance (player level)		0.017	<.001	0.014	<.001

^a^If the data belonged to the date before the participant played Pokémon GO, this value would be 0. Otherwise, it would be 1.

^b^If the data belonged to the date before the participant played Pokémon GO, this value would be 0. Otherwise, it would be the number of days he or she played.

^c^BMI: body mass index.

**Table 4:** Multilevel models of Pokémon GO and daily walking and running distances (km) models 3 and 4.

Variable				Model 3			Model 4			
				β		*P* value		β		*P* value
**Observation level**										
	**Pokémon GO or not^a^ (reference: not)**									
		Mean		0.093		<.001		0.072		.005
		Variance (player level)		0.018		<.001		0.017		<.001
		Covariance with intercept (player level)		−0.009		<.001		−0.008		<.001
	**Days played^b^**									
		Mean		−0.004		<.001		−0.005		<.001
		Variance (player level)		0.000096		<.001		0.000097		<.001
		Covariance with intercept (player level)		0.000068		.346		0.000059		.346
		Covariance with Pokémon GO (player level)		-0.00058		.004		−0.00057		.004
	**Weather**									
		Total rainfall		−0.001		.003		−0.001		.003
		Bright sunshine duration		0.003		.011		0.003		.012
		Mean air temperature		−0.013		.010		−0.013		.011
		Mean wind speed		−0.001		.037		−0.001		.036
**Players level**										
	**Age (reference >35)**									
		13-17		−0.068		.048		−0.067		.047
		18-23		−0.007		.414		−0.007		.415
		24-29		−0.010		.380		−0.009		.389
		30-35		−0.061		.031		−0.061		.031
	**Income (HKD; reference >30000)**									
		<5000		−0.024		.263		−0.024		.265
		5000-10000		−0.122		.005		−0.122		.005
		10001-15000		−0.041		.123		−0.040		.125
		15001-20000		0.044		.112		0.046		.103
		20001-30000		0.027		.239		0.028		.227
	**Gender (reference: female)**									
		Male		0.040		.019		0.041		.017
	**Education (reference: high school or lower)**									
		College or higher		−0.075		.037		−0.073		.040
	**BMI^c^ (reference: normal; kg/m^2^)**									
		Underweight (BMI<18.5)		−0.074		.002		−0.073		.002
		Overweight and obese (25≤BMI)		−0.052		.036		−0.051		.038
	**Built environment (reference: office)**									
		Mixed use area		0.032		.195		0.028		.191
		Green space		0.044		.068		0.001		.494
		Residential		−0.001		.490		−0.018		.309
		Retail premises		0.003		.460		0.001		.491
	**Interactions^d^ (reference: Pokémon GO x Office)**									
		Pokémon GO * Mixed use		­–		­–		0.006		.437
		Pokémon GO * Green space		–		–		0.068		.028
		Pokémon GO * Residential		–		–		0.027		.210
		Pokémon GO * Retail premises		–		–		−0.010		.406
**Intercept**										
	Mean		0.733		<.001		0.733		<.001	
	Variance (observation level)		0.053		<.001		0.053		<.001	
	Variance (player level)		0.016		<.001		0.016		<.001	

^a^If the data belonged to the date before the participant played Pokémon GO, this value would be 0. Otherwise, it would be 1.

^b^If the data belonged to the date before the participant played Pokémon GO, this value would be 0. Otherwise, it would be the number of days he or she played.

^c^BMI: body mass index.

^d^The value is equal to the value of Pokémon GO or not multiplied by built environment.

## Discussion

### Principal Results and Comparison With Prior Work

This study reported that after the game installation, average daily walking and running distances of Hong Kong Pokémon GO players increased by 18.1% (0.96 km，approximately 1200 steps) compared with the period before installation, which spanned for over a period of 35 days. These results were largely consistent with those of previous studies that were conducted in USA. For example, Howe et al found that the average number of daily steps for Pokémon GO players during the first week of installation increased by 955 steps [21]. Althoff and his colleagues indicated that Pokémon GO led to significant increases of more than 25% (1473 steps per day over a period of 30 days) [22]. These studies conducted in USA and the study conducted in Asia study showed that playing Pokémon GO was associated with an increase in physical activity in the first month players played the game. These results supported the idea that Pokémon GO may improve public health by promoting physical activity [32,33,38,39].

Our results showed that the increases in daily walking and running distances attenuated over time. This finding is also congruent with those of the previous studies conducted in USA [21,22]. However, the speed of attenuation was different between the study conducted by us, in Asia, and those conducted in USA. Although Howe et al [21] reported that the association was no longer observed after 6 weeks (42 days), our results indicated that the association disappeared faster, in 24 days, with Hong Kong players. This may be because Hong Kong is a relatively fast-paced society [40], which may make it easier for people to lose interest in the game. The high-density built environment, which lacks spacious places for playing location-based AR games, could also be another reason for this difference. If we wish to maximize the positive benefit from the game, initiatives should be taken to find ways to slow down attenuation.

From multilevel models, we found that total rainfall, bright sunshine duration, mean air temperature, and mean wind speed were significantly related to Pokémon GO players’ physical activity levels. The link between the weather and physical activity is well established. An increased duration of daily bright sunshine could increase daily walking activity [41] and participation in outdoor activities [42]; higher rainfall and wind speed are likely to be associated with lower physical activity levels [43-46]; the temperature could have different impacts on physical activities across areas and seasons [47]. For the season during which we conducted our study in Hong Kong, we found that the duration of bright sunshine was significantly associated with increased daily walking and running distances, whereas total rainfall, mean air temperature, and mean wind speed were negatively correlated with the distances. To the best of our knowledge, this is the first study that controlled the weather variables to provide a more precise relationship between Pokémon GO behaviors and physical activity levels. On December 7, 2017, the new version of Pokémon GO was introduced and included weather features, in addition to an in-game weather visual map, weather near Pokémon players will impact Pokémon in a variety of ways. This update made weather an even more important factor for the players [48].

The relationship between physical activity and built environment has been well investigated in recent decades [17]. As a location-based AR game, Pokémon GO is highly connected with built environment and both of these variables could be associated with players’ physical activity levels. In previous studies, however, built environment was not taken into consideration or they showed little impact on physical activity levels [21,22]. We examined Pokémon GO together with built environment and found that green space had a significant positive relationship with daily walking and running distances (Table 4). This result indicated that Pokémon GO may have encouraged players to use the green space around them to engage in physical activity. Nature-based recreation has decreased by 25% in the last 40 years [49], endangering the health benefits associated with nature and green space [50]. To solve this problem, Pokémon GO shows great potential to build connections between green space and opportunities for physical activity.

By controlling for the weather variable at the observation level and for demographics and built environment at the player level, our results showed that players with shorter daily walking and running distances exhibited stronger associations with Pokémon GO. These associations also lasted longer than players with relatively longer distances before installation. This result confirmed the previous findings from the studies conducted in USA that Pokémon GO is able to reach low activity populations [22,26]. Governments and public health agencies may consider the possibility of using location-based AR technology to improve physical activity and public health.

### Contributions

To the best of our knowledge, this study was one of the earliest and the only studies conducted during the Pokémon GO craze (from July 2016 to August 2016). This work offers 3 main contributions. First, we combined field surveys with automatically recorded physical activity data from mobile devices. The field study gave us an idea of who was playing the game outside and engaging in physical activity. Second, this paper was the first paper that investigated the relationship between Pokémon GO and physical activity with the weather and built environment added as covariates. Third, the difference in associations between Pokémon GO use and physical activity among different players was investigated. Such information is important to enhance the positive impact brought by future games or interventions. Further, as the first objective physical activity study of Pokémon GO in Asia, this study provided evidence together with other previous studies, which indicated that Pokémon GO was associated with short-term increases in physical activity levels.

### Limitations

Our study had some limitations. First, our study population was a representative of active Pokémon GO players; it was not a random sample of the Hong Kong population. It could not represent all the Pokémon GO players in Hong Kong. It is possible that some players may have only played at midnight (or during other irregular times), which was not within our survey time, or some people may have only played Pokémon GO in a place that was dissimilar to our study areas.

Second, this was a one group pretest-posttest design study. We acknowledge that we lack a control group; however, our data indicate that Pokémon GO alone was associated with physical activity changes in participants. During the study time, there was no campaign, parade, or other large-scale physical activity-related event that happened in Hong Kong. There were also no dramatic weather changes that could have led to increases in physical activity levels.

Further, we used daily walking and running distances as a proxy measurement of physical activity. We do acknowledge the possibility that other physical activities (eg, swimming and basketball) were likely to be not recorded if the players did not carry their phones during those activities.

### Conclusions

We studied 210 Pokémon GO players in Hong Kong during the Pokémon GO craze of 2016. Our results indicated that after the installation of the game, the average daily walking and running distances increased by 18.1% (0.96 km, approximately 1200 steps) compared with the levels measured before the installation. However, this association attenuated over time and was estimated to disappear after 24 days. The results of multilevel models indicated that the weather should be considered in this kind of research and the association between Pokémon GO use and physical activity was stronger among less physically active people compared with physically active people. We found that a game like Pokémon GO has the potential to build new links between humans and green space and to encourage people to engage in physical activity outdoor. Having a better understanding of the relationship between Pokémon GO use and physical activity may cast light on the future efforts needed to promote public health on a global scale.

## Abbreviations

BMI: body mass index
AR: augmented reality
